# Therapy-related myeloid neoplasms following peptide receptor radionuclide therapy for neuroendocrine neoplasms: case series reporting characteristics and outcomes from single-centre experience

**DOI:** 10.1530/EO-25-0107

**Published:** 2026-04-02

**Authors:** Wallace Chow, Ivy Wen, Claire Mok, Kathryn Huang, William Stevenson, Paul J Roach, Dale L Bailey, Connie I Diakos, Stephen J Clarke, Nick Pavlakis, David L Chan

**Affiliations:** ^1^ Department of Medical Oncology, Royal North Shore Hospital, Sydney, New South Wales, Australia; ^2^ Faculty of Medicine and Health, University of Sydney, Sydney, New South Wales, Australia; ^4^ Department of Haematology, Royal North Shore Hospital, Sydney, New South Wales, Australia; ^3^ Department of Nuclear Medicine, Royal North Shore Hospital, Sydney, New South Wales, Australia

**Keywords:** peptide receptor radionuclide therapy, neuroendocrine neoplasm, leukaemia, myeloid neoplasm

## Abstract

**Objective:**

Peptide receptor radionuclide therapy (PRRT) with [177Lu]Lu-DOTATATE is increasingly used in the treatment of metastatic neuroendocrine neoplasms (NENs). Marrow toxicity resulting in therapy-related myeloid neoplasms (t-MNs) remains a rare but fatal complication of PRRT, with a limited understanding of prognostic or predictive factors.

**Methods:**

We conducted a single-centre retrospective review of all patients with metastatic NEN who received at least one cycle of PRRT and subsequently developed t-MN confirmed on bone marrow biopsy.

**Results:**

Thirteen out of 306 patients (4.2%) developed t-MN during follow-up, confirmed on bone marrow biopsy. The median time from cycle 1 of PRRT to diagnosis of t-MN was 48.3 months (range: 5.4–110.1 months). The median number of PRRT cycles was 4 (range: 3–8). Nine (69%) patients received concomitant radiosensitising chemotherapy with capecitabine, and one (7.7%) had the combination of capecitabine and temozolomide. The median overall survival from cycle 1 of PRRT was 61.9 months (range: 18.5–112.2 months). The median overall survival from diagnosis of t-MN was 10.4 months (range: 1.9–89.3 months). An unfavourable karyotype, a higher degree of cytopaenia and a higher blast percentage in bone marrow were associated with worse survival outcomes of t-MN.

**Conclusions:**

t-MN following PRRT is an uncommon but serious complication that can occur several years after treatment. This case series highlights the poor prognosis following t-MN diagnosis, particularly if untreated. PRRT is an effective treatment modality for NEN, but prospective studies are needed to explore factors predictive of t-MN development.

## Introduction

Peptide receptor radionuclide therapy (PRRT) with [177Lu]Lu-DOTATATE is increasingly used in the treatment of unresectable metastatic neuroendocrine neoplasms (NENs) that express somatostatin receptors (1, 2). PRRT is generally well tolerated with limited acute and medium-term toxicities. However, PRRT is associated with the long-term toxicities of renal and bone marrow impairment. Whilst the risk of renal toxicity can be reduced by the infusion of amino acids with PRRT (3, 4), there are currently no effective strategies to reduce the risk of bone marrow toxicity.

The reporting of incidence and outcomes of therapy-related myeloid neoplasms (t-MNs), which encompasses myelodysplastic syndrome (MDS) and acute myeloid leukaemia (AML), is limited with a reported incidence rate of 1–5.4% (5, 6, 7, 8). Single-centre series studies have reported that patients that develop t-MN after PRRT had a median overall survival of 13 months from t-MN diagnosis, which is substantially reduced from the median overall survival of 59–62 months from cycle 1 of PRRT (6). AML, the most feared complication of PRRT, occurs in up to 2% of patients and portends a median survival of 7 months from diagnosis (6).

Whilst these studies attempted to identify potential negative prognostic factors, including acute thrombocytopaenia, unfavourable cytogenetic mutations and prior chemotherapy exposure, study numbers were limited (6). In the current article, we present our t-MN experience from a quaternary referral academic NEN centre (Royal North Shore Hospital, Sydney, Australia). Potential PRRT candidates are carefully selected after multidisciplinary review to ensure suitability for treatment. We conducted a retrospective study of NEN patients treated with PRRT at our centre to determine the incidence of t-MN, survival outcomes and potential predictive factors for t-MN development.

## Method

A retrospective review was performed of all NEN patients treated with at least one cycle of PRRT at our centre, through reviewing the NEN patient database, with the index date (date of first PRRT administration) from August 2013 to October 2024. Included patients had a histologically confirmed, advanced (unresectable and/or metastatic) NEN. Review of medical records and pathology was conducted in August 2025 to identify cases with MDS or AML confirmed through bone marrow biopsy. Further information on patient and NEN characteristics, t-MN characteristics, treatments received and survival outcomes was collected through chart review and reported descriptively. Given the small number of patients in this study, a formal comparison between subgroups was not performed. Ethics approval was sought from Northern Sydney Local Health District Human Research Ethics Committee (2024/ETH02131), and the study was deemed eligible for a HREC approval exemption as a case series.

## Results

### Clinicopathological characteristics

A total of 306 patients with NEN were treated with at least one cycle of PRRT between August 2013 and October 2024. Thirteen patients (4.2%) developed t-MN during follow-up, confirmed on bone marrow biopsy. Median follow-up time from cycle 1 of PRRT was 61.9 months (range: 18.5–112.2 months).

Baseline characteristics of the thirteen patients are shown in Table 1. Eight patients (62%) were female with a median age of 65.3 years (range: 42.8–75.0 years) at NEN diagnosis. Most patients had disease arising from small bowel (62%) or pancreas origin (23%), with the other two patients having disease arising from appendix and breast. Six of 13 (46%) were WHO grade 1, six (46%) were grade 2, and one (8%) was grade 3. The most common sites of metastases were the liver (92%), lymph nodes (62%) and bone (38%). Twelve of 13 (92%) patients had more than five metastases prior to C1 PRRT.

**Table 1:** Patient and NEN characteristics.

Case	Age at NEN diagnosis (years)	Gender	Primary site	WHO grade 2022	Ki-67 index	Sites of metastases at diagnosis	Number of metastases prior to C1 PRRT
1	72.7	Female	Small bowel	2	10	Liver, lymph nodes	>5
2	68.1	Male	Pancreas	1	<1	Liver, lymph nodes	>5
3	51.2	Female	Small bowel	1	1	Liver, lymph nodes	>5
4	54.2	Male	Small bowel	1	<1	Liver, lymph nodes, bone, lung, peritoneum	>5
5	55.2	Male	Appendiceal	1	<1	Liver, pancreas	>5
6	65.3	Female	Small bowel	2	10	Liver, bones	>5
7	67.5	Female	Small bowel	1	2	Liver, lymph nodes, bones	>5
8	59.5	Male	Pancreas	2	10	Liver, bone, lung	>5
9	73.8	Female	Breast	3	90	Lymph nodes, bone	2
10	61.1	Female	Small bowel	2	-	Liver, lymph nodes, peritoneum	>5
11	75.0	Female	Small bowel	1	1	Liver, vaginal vault, peritoneum, stomach, splenic hilum	>5
12	42.8	Female	Pancreas	2	12	Liver	>5
13	67.1	Male	Small bowel	2	7	Liver, lymph nodes	>5

### PRRT/NEN treatment characteristics

Treatment parameters for NEN are shown in Table 2. Prior to PRRT, the majority of patients received somatostatin analogues (92%), and this was the only prior treatment for three patients (23%). Over half of patients (62%) had prior resection of primary site of disease. Only three patients (23%) received chemotherapy prior to PRRT, with one of the three receiving multiple lines of chemotherapy (CAPTEM, raltitrexed/oxaliplatin and carboplatin/etoposide). All patients received PRRT for radiological disease progression, with one patient also experiencing uncontrolled hormone-related symptoms. The median time from NEN diagnosis to cycle 1 of PRRT was 30.5 months (range: 5.5–139.2 months). The median number of PRRT cycles was 4 (range: 3–8), with five patients (38%) being re-treated with PRRT after further progression. Nine (69%) patients received concomitant radiosensitising chemotherapy with oral capecitabine, and one (7.7%) had oral capecitabine in combination with temozolomide. At 3 months after the start of PRRT, most patients had either NEN disease stabilisation (*n* = 5, 38%) or partial response (*n* = 6, 46%), with two (15%) demonstrating disease progression. Regarding subsequent therapies, nine (69%) patients continued somatostatin analogues after PRRT, five (38%) patients received liver-directed therapy, and one patient received further chemotherapy with capecitabine. Acute cytopaenia after PRRT (defined as within 8 weeks after each cycle PRRT treatment) occurred in ten patients (77%). Grade 3 cytopaenia occurred in four patients, grade 2 cytopaenia occurred in one patient, and grade 1 cytopaenia occurred in five patients, graded according to the Common Terminology Criteria for Adverse Events (CTCAE), version 5.0 (9). Thrombocytopaenia was the most common acute cytopaenia observed following PRRT (nine patients), followed by neutropaenia (four patients) and anaemia (five patients).

**Table 2:** PRRT + NEN treatment characteristics.

Case	Pre-PRRT treatment	Number of lines of prior to systemic therapy	Treatment indications	Timing of first-cycle PRRT from NEN diagnosis (months)	Number of cycles of PRRT	Concurrent chemotherapy	Acute cytopaenia post-PRRT	Post-PRRT treatment	Progression-free survival (months)
1	SSA	1	PD	24.7	4	Cape ×4	Nil	SSA, LDT, others	19.3
2	SSA	1	PD	30.5	3	Nil	G3 thrombocytopaenia	Nil	7.7
3	SSA, surgery	1	PD	53.4	8	CAPTEM ×4, cape ×4	G1 thrombocytopaenia	Nil	46.5
							G1 anaemia		
4	SSA, surgery	1	PD	66.9	4	Cape ×4	Nil	SSA	69.8
5	SSA, surgery, chemotherapy (fluorouracil), LDT	2	PD	139.2	8	cape ×4	G1 thrombocytopaenia	SSA, LDT	32.9
							G1 anaemia		
							G3 neutropaenia		
6	SSA	1	PD	14.9	6	Cape ×2	G3 thrombocytopaenia	SSA, chemotherapy (capecitabine), LDT	39.6
							G3 neutropaenia		
7	SSA, surgery, radiotherapy	1	PD	116.9	6	Nil	G3 thrombocytopaenia	SSA	39.5
							G2 anaemia		
							G1 neutropaenia		
8	SSA, chemotherapy (capecitabine/temozolomide)	2	PD	54.4	8	cape ×4	G1 thrombocytopaenia	SSA, LDT	22.6
							G1 anaemia		
							G1 neutropaenia		
9	Surgery, others	0	PD	5.5	3	Nil	Nil	Surgery, others	3.6
10	SSA, surgery, LDT	1	PD, symptoms	32.3	4	Cape ×1	G2 thrombocytopaenia	SSA, LDT	50.6
11	SSA, surgery	1	PD	8.9	4	cape ×2	G1 thrombocytopaenia	SSA	18.4
12	SSA, chemotherapy (capecitabine/temozolomide, raltitrexed/oxaliplatin, carboplatin/etoposide), LDT	4	PD	27.9	3	Cape ×1	G1 thrombocytopaenia	Surgery, others	7.2
							G1 anaemia		
13	SSA, surgery	1	PD	29.6	4	Nil	G1 anaemia	SSA	18.5

Cape, capecitabine; CAPTEM, capecitabine and temozolomide; LDT, liver-directed therapy; SSAs, somatostatin analogues; PD, progression of disease.

### t-MN diagnosis/treatment characteristics

**Figure 1:**
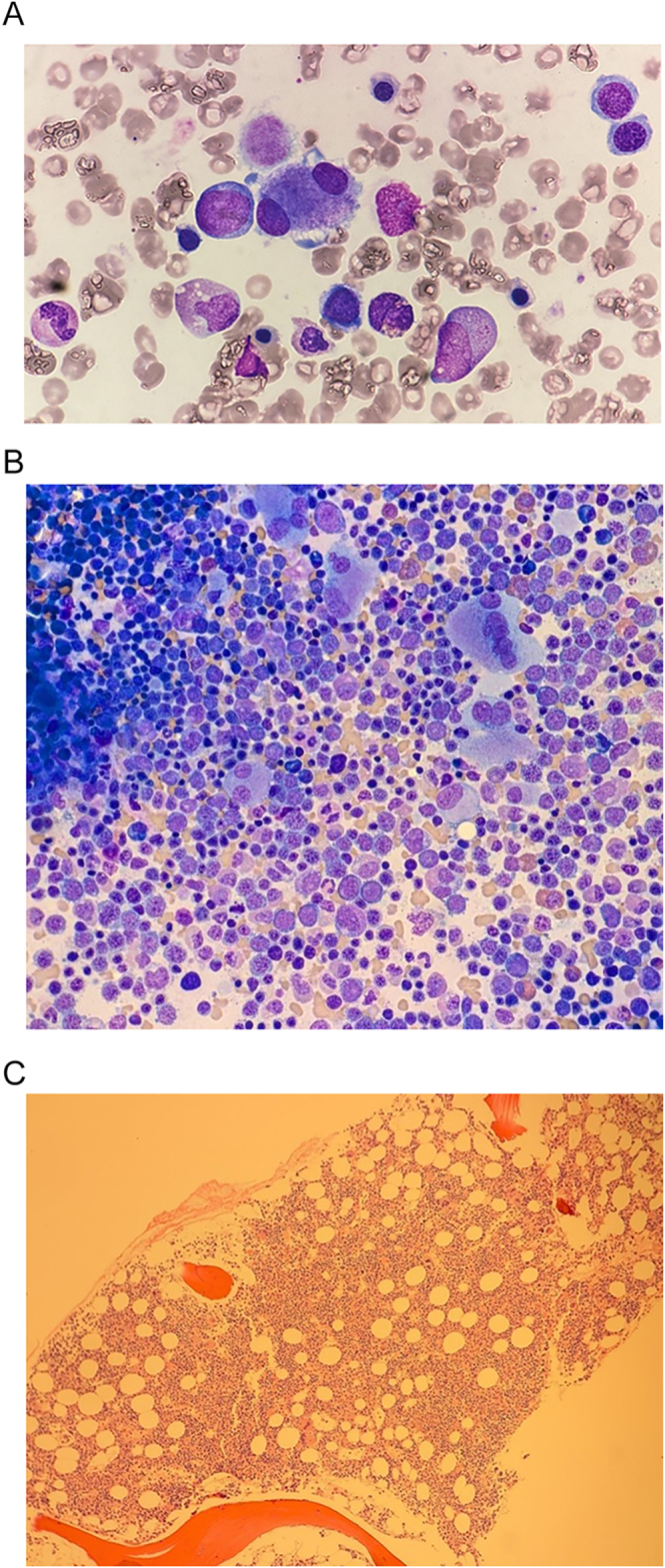
Bone marrow biopsy of case 4 with MDS. (A) Bone marrow aspirate 100x (top image) – marked dysmegakaryopoiesis with a small hypolobulated megakaryocyte demonstrated with two separate nuclei at opposite poles of the cell. Dyserythropoiesis with irregular nuclear contours and increased karyorrhexis. Vacuolated granulocytic precursors. (B) Bone marrow aspirate 40× (bottom left) – increased erythroid and megakaryocytic activity with significant dysmegakaryopoiesis and dyserythropoiesis. (C) Bone marrow trephine haematoxylin & eosin, 10× (bottom right) – marked hypercellularity with increased erythroid and megakaryocytic activity and left-shifted granulopoiesis. Clustering of megakaryocytes. Diffusely increased reticulin deposition.

All patients had bone marrow biopsy confirmation of t-MN for the investigation of persistent cytopaenia, with characteristic features as seen in patient case 4 (Fig. 1). Ten patients were diagnosed with MDS, and three patients were diagnosed with AML. None of the patients diagnosed with MDS had developed AML at the time of follow-up. The median time from cycle 1 of initial PRRT to the diagnosis of t-MN was 48.3 months (range: 5.4–110.1 months). At the time of t-MN diagnosis, four (31%) patients continued to have stable disease after PRRT, with only somatostatin analogues as subsequent therapy, whilst the other nine patients had progressed. Karyotype analysis was available for twelve patients, with seven (54%) demonstrating complex unfavourable karyotype conferring a poorer risk (10), detailed in Table 3. Next-generation sequencing results were available for six patients, with the most common molecular mutations found in TP53 (*n* = 3) and TET2 (*n* = 3). Ten patients (77%) had anaemia at diagnosis (defined by haemoglobin <100 g/L), ten patients (77%) had thrombocytopaenia (defined by platelet count <100 × 10^9^/L), and five patients (38%) had neutropaenia (defined by absolute neutrophil count <1.0 × 10^9^/L). The median blast percentage on bone marrow biopsy was 1% (range: 1–4%) for MDS diagnosis and 30% (range: 29–39%) for AML diagnosis.

**Table 3:** t-MN and t-MN treatment characteristics and outcomes.

Case	Timing of t-MN diagnosis after first-cycle PRRT (months)	Diagnosis	Karyotype	Karyotype risk	Next-generation sequencing panel mutations	Haemoglobin at t-MN diagnosis (g/L)	Absolute neutrophil count at t-MN diagnosis (×10^9/L)	Platelet count at t-MN diagnosis (×10^9/L)	Bone marrow blasts (%)	t-MN treatment	Status	Survival after t-MN diagnosis (months)	Overall survival after cycle 1 PRRT (months)	Cause of death
1	110.1	MDS	45,XX,-7[8]/45,idem,del(12)(p11.2)[4]	Poor	N/A	78	0.6	53	4	Decitabine/cedazuridine	Deceased	2.1	112.2	t-MN
2	5.4	MDS-SLD	Normal	Good	N/A	117	1.4	30	1	Nil	Alive	89.3	94.8	-
3	83.4	AML	44∼46,XX,der(3)t(3;17)(p12;q11.2),add(3)(p12),del(5)(q31q32),-7,+8,der(12)t(12;13)(p11.2;q12),add(12)(p13),-13,-14,-16,-17,+1∼4mar/46XX[3]	Poor	2x somatic TP53 variants (C277Y, L130P)	73	0.3	34	28	Azacitidine/venetoclax	Alive	10.4	93.8	-
4	40.9	MDS-RS-MLD	43,XY,der(3)(??),der(5)?ins(5)(q31),-6,-7,der(12)(?p13),der(14)(?q32), -17,-19,+mar[cp6]/46,XY[4]	Poor	Somatic TP53 (C1024T), somatic TET2 (846dup)	85	1.1	49	4	Azacitidine, alloSCT	Alive	29.0	69.8	-
5	48.3	Smouldering IgG myeloma + MDS	Normal	Good	Somatic TET2 (G3890A)	111	0.7	149	1	Nil	Deceased	19.8	68.1	NEN
6	61.3	MDS	Normal	Good	N/A	85	1.1	111	1	Nil	Deceased	5.4	66.7	NEN
7	60.0	AML	45, XX, -7[1]/45,sl,add(3)(q21),add(8)(q24),+mar[8]/45,sdl,der(X)add(X)(p11.2-11.4)del(X)(q21-24q28)[7]/45,sdl,add(6)(q13)[3]/45,sdl,del(16)(q12-13q24)[5]	Poor	PPM1D (1619del)	90	0.5	42	30	Nil	Deceased	1.9	61.9	t-MN
8	55.8	MDS	N/A	-	N/A	86	0.8	95	-		Deceased	1.9	57.7	t-MN
9	49.7	AML	46∼48,XX,del(5)(q31),der(7)t(7;12)(q11.2;q13),der(11)hsr(q13),-12,-13,+3∼5mar[20]	Poor	TP53 (G711A), IDH2 (A505G)	85	1.3	26	39	Nil	Deceased	4.2	53.9	t-MN
10	34.8	MDS-RS-MLD	45,XX,t(4;10)?(q12;q26),der(6)(p22),-8,-18,+mar[13]/46,XX[7]	Poor	N/A	102	1.2	33	2	Azacitidine	Deceased	15.8	50.6	t-MN
11	18.4	MDS	arr(X,1-22)x2	Intermediate	DNMT3A, EZH2, 3x PPM1D and 2x TET2 (variant info N/A)	89	1.7	136	1	Nil	Alive	11.9	30.4	-
12	8.5	MDS	Normal	Good	N/A	89	7.3	73	1	Nil	Deceased	11.5	20.0	NEN
13	8.6	MDS-RS-MLD	45,XY,-5,der(7)del(7)(q22)?t(7;10)(q22;q22),+8,-10,t(14;16)(q12;q22), der(17)(p11.2)[18]/46,XY[2]	Poor	N/A	87	2.2	37	4	Azacitidine	Deceased	9.9	18.5	t-MN

MDS-SLD, myelodysplastic syndrome with single-lineage dysplasia; MDS-RS-MLD, myelodysplastic syndrome with ring sideroblasts and multilineage dysplasia; and N/A, not avail.

Eight (62%) patients did not receive any treatment for t-MN, either due to frailty or due to indolent disease managed with transfusion support. Three (23%) patients received azacitidine, one followed by allogeneic stem cell transplant. One patient received azacitidine and venetoclax, and one received decitabine and cedazuridine.

### Overall survival and follow-up

**Figure 2:**
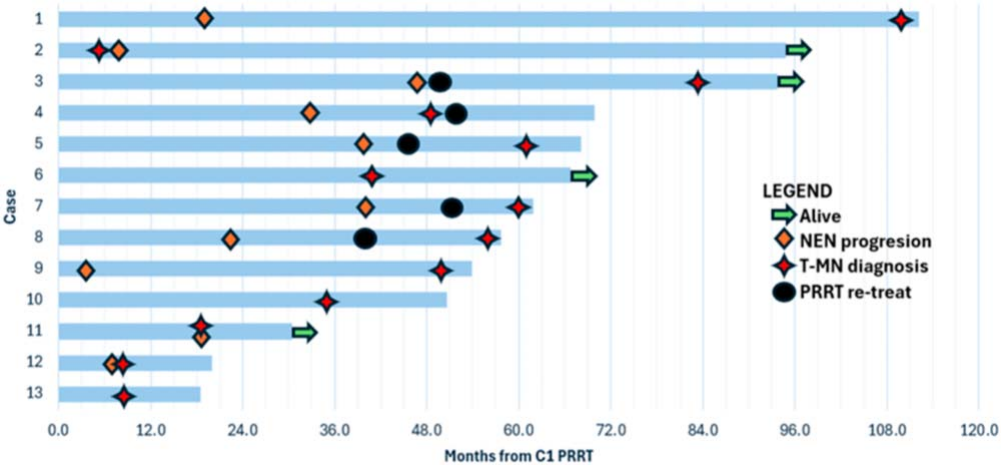
Swimmer plot of patients from cycle 1 PRRT.

Four of thirteen (31%) patients remain alive at the time of analysis. The median overall survival from cycle 1 of PRRT was 61.9 months (range: 18.5–112.2 months). The median overall survival from the diagnosis of t-MN was 10.4 months (range: 1.9–89.3 months). Of the nine patients that died, six (46%) died due to t-MN or sepsis as a complication of t-MN and three (23%) died due to progressive disease. Of the four patients that remain alive: one remains in remission following allogeneic stem cell transplant for MDS 29.0 months since the diagnosis of t-MN; one continues on supportive therapy for low-risk MDS after 89.3 months; one continues on azacitidine and venetoclax for AML 10.4 months since diagnosis; and one continues on supportive therapy for MDS 11.9 months since diagnosis. A swimmer plot of patient timelines from cycle 1 of PRRT is depicted in Fig. 2.

In our cohort, the median overall survival from t-MN diagnosis of patients with an unfavourable karyotype (*n* = 7) was 9.9 months (range: 1.9–29.0 months) and that of patients with a favourable karyotype (*n* = 4) was 15.7 months (range: 5.4–89.3 months). A higher degree of cytopaenia (2 or 3 cell lineages versus 0 or 1 cell lineage affected) was associated with worse outcomes, with a median overall survival from t-MN diagnosis of 7.0 months (range: 1.9–29.0 months, *n* = 8) compared to 15.8 months (range: 5.4–89.3 months, *n* = 5). Patients with blast percentage in bone marrow of 2% or less had better outcomes than those with 3% or higher, with a median overall survival from t-MN diagnosis of 15.9 months (range: 5.4–89.3 months, *n* = 6) compared to 7.0 months (1.9–29.0 months, *n* = 6).

## Discussion

PRRT is increasingly utilised as a standard-of-care treatment for patients with progressive NEN disease, with growing evidence of efficacy (1, 2, 11). This retrospective analysis highlights the uncommon but serious risk of marrow toxicity in the form of MDS or AML following PRRT. Our single-centre experience over the past decade of t-MN following PRRT is in keeping with previously reported incidences at 4.2% (6, 7).

**Figure 3:**
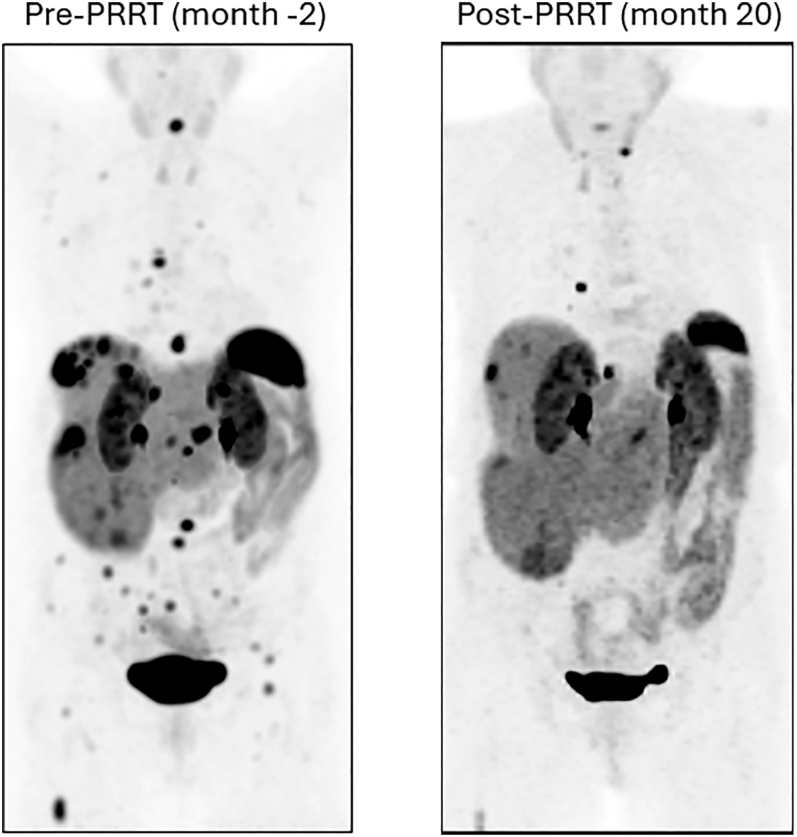
68Ga-DOTATATE PET images of pre-PRRT (left image) and post-PRRT (right image) with [177Lu]Lu-DOTATATE in case 7, maximum intensity projection images.

The median overall survival from cycle 1 of PRRT of 61.9 months was similar to previously reported survival rates. However, the median overall survival from t-MN diagnosis was only 10.4 months, also similar to previous Australian reports (6). In our series, 31% of cases had not progressed from their NEN disease at the time of t-MN diagnosis, highlighting the rare but troubling consequences of an otherwise effective treatment modality of PRRT for NEN. Therefore, whilst many patients have a good and durable clinical response to PRRT, evidenced by a radiological improvement on 68Ga-DOTATATE PET (Fig. 3), this comes at a potential cost of developing secondary haematological malignancy. Our series of patients also confirms established poor prognostic variables in t-MN, including an unfavourable karyotype, a higher degree of cytopaenia and a higher percentage of blasts in bone marrow biopsy (6, 10).

The majority of our cases received radiosensitising capecitabine as part of our institutional protocol, but its contribution to the development of t-MN is questioned as our incidence rate is similar to other published series (6, 7). There was only one case identified with t-MN that had received radiosensitising chemotherapy with combined capecitabine and temozolomide. Our institution has had increasing use of CAPTEM given recently published evidence that PRRT combined with CAPTEM has a higher response rate and progression-free survival without added late haematological toxicities (12, 13). Otherwise, only four patients (31%) were treated with lines of chemotherapy before or after PRRT, demonstrating a limited relationship between chemotherapy use and the development of t-MN.

The majority of patients (92%) had a tumour burden of greater than five metastases, although this may be more reflective of the patient selection for PRRT treatment rather than a predictive factor for developing t-MN. Multiple sites of metastases is often more appropriate for PRRT than lower volume, oligometastatic disease that could be targeted with local therapies or somatostatin analogues alone. Future studies could further assess for any relationship between tumour volume and burden, particularly in bones, and developing t-MN, although this was not shown to be a risk factor previously (6).

Acute haematological toxicities, particularly thrombocytopaenia, have also been proposed as a possible risk factor (6). In our case series, acute cytopaenias were first detected during PRRT in the majority of cases, again with thrombocytopaenia as the most common cytopaenia that may be a predictive factor for developing t-MN. The presence of clonal haematopoiesis of indeterminate potential (CHIP), somatic mutations found in blood or marrow cells that may act as precursors to myeloid neoplasms (14), is a growing area of study. A prospective study of 37 patients with metastatic NEN treated with PRRT found that the prevalence of CHIP mutations was 35%, and this is associated with acute thrombocytopaenia risk after PRRT for NEN, but not anaemia and neutropaenia (15). The most common somatic mutations seen were variants in TET2, DNMT3A and ASXL1 as commonly seen in the general ageing population. Pathogenic mutations in DNA damage response genes, such as PPM1D, ATM2, TP53 and CHEK2, have increased incidence after exposure to chemotherapy and/or radiotherapy and are also more prevalent in t-MN compared to primary *de novo* myeloid neoplasms (16, 17, 18). Future studies may help determine whether the presence of these CHIP mutations may be relevant as a predictor for a higher risk of developing t-MN, or if next-generation sequencing myeloid panels on peripheral blood could be used to screen for t-MN in patients with cytopaenia without formal bone marrow biopsy.

Pre-clinical studies have shown expression of somatostatin receptor 2 (SSTR2) in haematopoietic cells, specifically in IL-3-dependent murine myeloid cell lineage, providing a potential biological mechanism of action for t-MN development (19). Furthermore, Nguyen and colleagues have recently demonstrated that haematopoietic stem cells and multipotent progenitor cells showed not only similar SSTR2 expression to neuroendocrine tumour cells but increased binding to ^177^Lu-labelled SSTR2 antagonists causing reduced proliferation to further strengthen this hypothesis (20). Five patients were re-treated with PRRT, which could lead to an increased risk of developing t-MN as previously suggested as a result of cumulative myelotoxicity (21, 22). A higher-grade of NEN disease does not appear to confer risk of developing t-MN, with 46% patients having grade 1 disease on initial histopathology.

In terms of treatment for t-MN, one patient was successfully treated with azacitidine and venetoclax, followed by allogeneic stem cell transplant, and remains in remission of both NEN and t-MN at 29.0 months since t-MN diagnosis. One patient developed low-risk MDS of single lineage, without treatment besides infrequent transfusions, and remains alive 89.3 months since diagnosis. These two cases demonstrate the potential to treat t-MN with success, possibly limited by patient fitness for treatment, t-MN disease risk and state of NEN disease.

A limitation is the retrospective nature of our study. Given that our centre acts as a quaternary referral centre receiving referrals from across our state for PRRT, we acknowledge a potential risk of under-identification from patients lost to follow-up. Another limitation is the invasive nature of diagnosis via bone marrow biopsy that can be practically challenging or unsuitable for certain patients. During retrospective review, sixteen patients (5.2%) in our cohort were identified to have persistent cytopaenias or had regular blood transfusion requirements that raise the suspicion of t-MN, but were not confirmed on bone marrow biopsy. Whilst there are confounding factors that will account for these findings other than t-MN, there is the possibility that the risk of t-MN is underappreciated. The small number of patients in this study also limited formal statistical and multivariate analysis.

## Conclusion

This single-centre experience of PRRT for NEN details the characteristics and outcomes of t-MN following PRRT. Whilst PRRT remains a highly effective therapy for treating metastatic NEN, survival outcomes are substantially reduced if patients develop t-MN. Further prospective studies are needed to better understand prognostic and predictive factors for developing t-MN and thus minimise risk and improve patient selection when assessing for PRRT suitability.

## Declaration of interest

The authors declare that there is no conflict of interest that could be perceived as prejudicing the impartiality of the work reported.

## Funding

DLC was supported by Early Career Fellowship from Cancer Institute NSW.

## Author contribution statement

WC collected data and wrote manuscript; IW, CM, KH, WS, PR, DB, CD, SC and NP reviewed and edited manuscript; and DC conceived the study, is the primary supervisor and reviewed and edited manuscript.
